# Surgical Resection with Neoadjuvant Chemotherapy for Locoregionally Recurrent Appendiceal Cancer Invading the External Iliac Vessels

**DOI:** 10.1155/2018/1674279

**Published:** 2018-08-02

**Authors:** Jun Takahashi, Shingo Tsujinaka, Nao Kakizawa, Noriya Takayama, Erika Machida, Kazuki Iseya, Fumi Hasegawa, Rina Kikugawa, Yasuyuki Miyakura, Koichi Suzuki, Toshiki Rikiyama

**Affiliations:** Department of Surgery, Saitama Medical Center, Jichi Medical University, 1-847, Amanumacho, Omiya, Saitama-shi, Saitama 330-8503, Japan

## Abstract

Recent advancements in multimodal therapy can provide oncologic benefits for patients with recurrent colorectal cancer. This report presents a case of locoregionally recurrent appendiceal cancer treated with neoadjuvant chemotherapy followed by surgical resection with vascular reconstruction. A 68-year-old Japanese woman was diagnosed with appendiceal cancer and underwent ileocecal resection. The pathological evaluation revealed KRAS-mutant adenocarcinoma with the final stage of T4bN1M0. She received oral fluorouracil-based adjuvant chemotherapy. One year later, she was found to have peritoneal dissemination in the pelvic cavity and vaginal metastasis. She received an oxaliplatin-based chemotherapy followed by surgical resection. One year after the second surgery, she developed a locoregional recurrence involving the right external iliac vessels and small intestine. She received an irinotecan-based regimen with bevacizumab as neoadjuvant chemotherapy, followed by surgical resection. At first, a femoro-femoral bypass was made to secure the blood supply to the right lower extremities. Subsequently, an en bloc resection including the recurrent tumor and the external iliac vessels was completed. Surgical resection for recurrent colorectal cancer is often technically challenging because of the tumor location and invasion to adjacent organs. In this case, a surgical approach with persistent chemotherapy achieved oncologic resection of locoregionally recurrent appendiceal cancer.

## 1. Introduction

The recurrence rate following curative resection with adjuvant chemotherapy for stage II or III colorectal cancer is approximately 30% [1]. Locoregional recurrence after curative resection for colon cancer is uncommon, with a reported incidence of 4.4% to 12.8% [2–5]. Although previous studies have demonstrated that complete surgical resection improved survival for locoregionally recurrent colon cancer (LRCC) [5–8], curative surgery is often challenging because it may lead to complex multivisceral resection. Neoadjuvant chemotherapy and/or radiotherapy may be considered for LRCC; however, little evidence is available due to a lack of prospective trials. Hence, we herein report a case of locoregionally recurrent appendiceal cancer treated by surgical resection with neoadjuvant chemotherapy.

## 2. Case Presentation

A 68-year-old Japanese woman with no significant medical history had suffered from pain in her right lower quadrant. She was examined at the regional cancer center and diagnosed with appendiceal cancer. She underwent ileocecal resection with lymph node dissection at the aforementioned hospital, and the postoperative course was uneventful. The pathological evaluation revealed KRAS-mutant (codon 12) moderately to poorly differentiated adenocarcinoma, and the final TNM stage was T4b (small intestine) N1M0 (stage IIIc). Thereafter, she received adjuvant chemotherapy with oral tegafur/uracil and leucovorin for one year.

One year after the surgery, a positron emission tomography/computed tomography (PET/CT) scan showed sporadic foci of intense tracer uptake in the pelvic cavity, consistent with peritoneal dissemination and vaginal metastasis. The recurrent tumor deposits were considered resectable. She received three courses of mFOLFOX6 regimen (oxaliplatin, folinic acid, and fluorouracil) as neoadjuvant chemotherapy, followed by resection of the peritoneal dissemination and partial resection of the vagina. The pathological diagnosis confirmed negative resection margins. Then, she resumed the mFOLFOX6 regimen as adjuvant chemotherapy; however, the regimen was discontinued after two courses for an allergic response to oxaliplatin.

**Figure 1:**
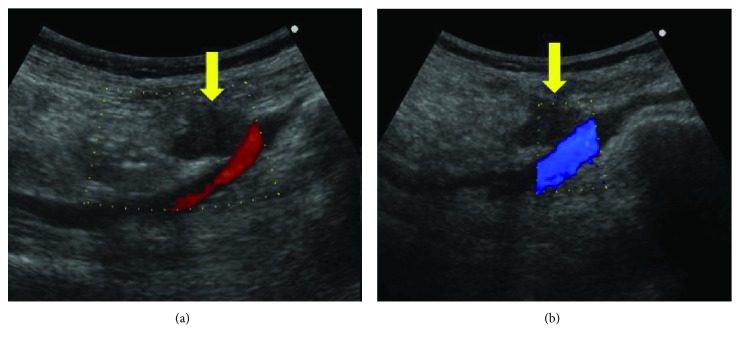
Ultrasonography showing a 27 × 16 mm, irregular, low-echoic tumor (arrow). The tumor is located around the external iliac artery (a) and the external iliac vein (b).

**Figure 2:**
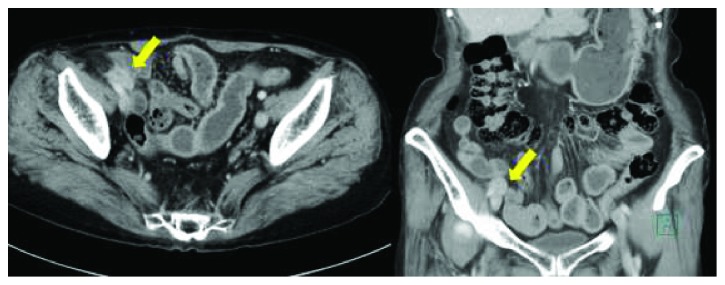
Contrast-enhanced CT scan showing an irregular tumor around the right external iliac vessels and the small intestine, contiguous to the small intestine (arrow).

**Figure 3:**
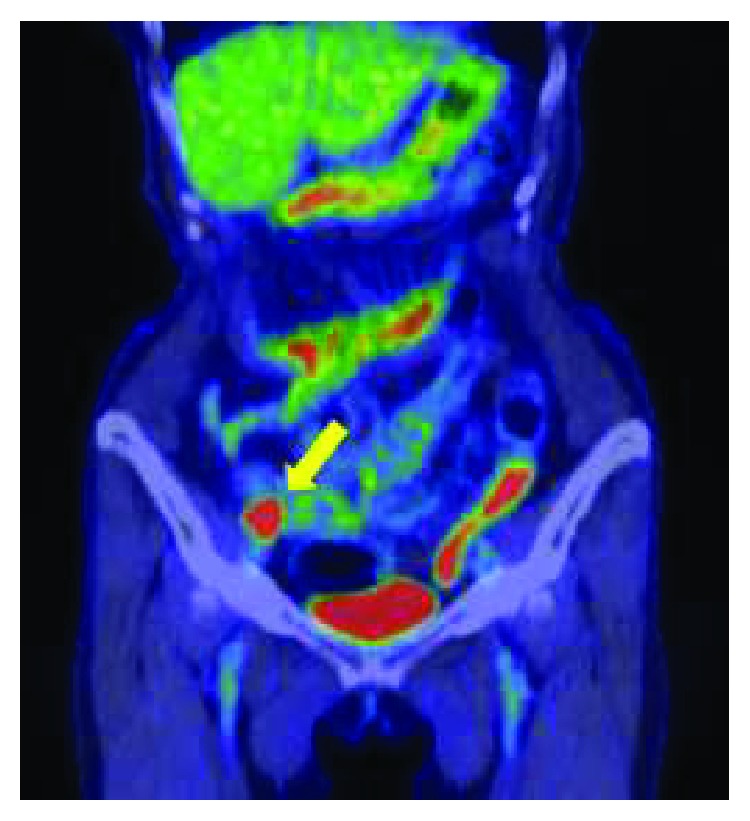
PET-CT showing intense tracer uptake (SUV max: 17.5) at the tumor (arrow).

One year after the second surgery, a surveillance abdominal ultrasonography showed a 27 × 16 mm irregular and low-echoic tumor around the right external iliac artery (Figure 1). A contrast-enhanced CT scan showed an irregularly enhanced tumor around the right external iliac artery and vein. In addition, the tumor appeared contiguous to the small intestine. These findings suggested tumor invasion to the right external iliac artery, the right external iliac vein, and the small intestine (Figure 2). A PET/CT scan showed tracer uptake (standardized uptake value max: 17.5) at the tumor (Figure 3). It also showed regional lymph node enlargement around the right iliac vessels and no findings of distant metastases. The patient was diagnosed with a locoregional recurrence after the first reoperative surgery for appendiceal cancer. She was then referred to our hospital for potential surgical resection.

We assumed that the tumor was resectable; however, the external iliac vessels also required resection during the surgery, which required a simultaneous procedure for vascular reconstruction. We proposed neoadjuvant chemotherapy and subsequent surgery for the patient because it was deemed important to control the tumor progression and increase tumor resectability.

We selected a FOLFIRI (irinotecan, folinic acid, and fluorouracil) plus bevacizumab regimen as neoadjuvant chemotherapy, considering the mutant KRAS status. We initially planned six courses of FOLFIRI plus bevacizumab therapy and assess the tumor progression and resectability every 3 months. We discussed with the patient, and she agreed with our proposed treatment strategy. Follow-up CT scans after three and six treatment courses showed no tumor progression. After six courses of the treatment, we recommended surgical resection for her as we planned, but she wanted to receive chemotherapy. FOLFIRI plus bevacizumab regimen was continued accordingly.

After eight courses, she developed edema in the right lower extremities. A contrast-enhanced CT showed a narrowing of the right external iliac vein without any thromboemboli in both lower extremities; however, she was incidentally found to have suspicious emboli in her pulmonary arteries. The neoadjuvant chemotherapy was terminated, and she was administered an anticoagulant therapy (edoxaban). After two months, a contrast-enhanced CT was taken again and showed no thromboemboli and no significant tumor progression. Although she had been asymptomatic for the suspicious pulmonary embolism, her lower extremities remained edematous. We, thus, indicated surgical resection of the tumor. The tumor was considered to be resectable, and we asked cardiovascular surgeons for vascular reconstruction at the time of surgery.

**Figure 4:**
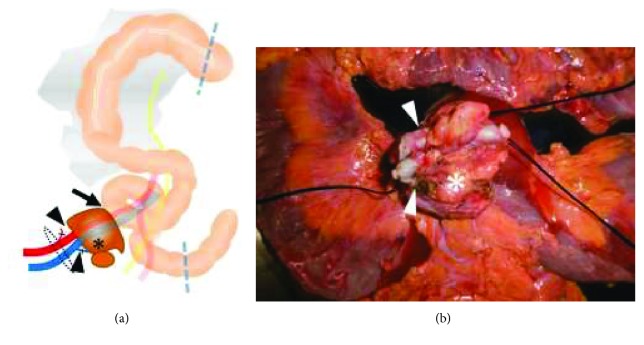
A femoro-femoral arterial bypass was performed, followed by an en bloc tumor resection. (a) The fixed solid tumor (^∗^) at the right inguinal fossa. The tumor involved the external iliac artery, external iliac vein (arrowhead), ileum (arrow), and right obturator lymph nodes. The broken lines with blue color indicate the proximal and distal resection margins of the bowel. The broken lines with black-colored circle indicate the resection margin of the vessels. (b) Surgical specimen of the resection tumor (^∗^) that invaded, including both the external iliac artery and vein (arrowhead) as well as the small intestine.

The surgery was performed in the following sequence. At first, vascular reconstruction was performed by cardiovascular surgeons. A femoro-femoral arterial bypass with synthetic vascular graft (Gelsoft™ 8 mm, Vascutek Ltd., Renfrewshire, Scotland, UK) was made to secure the blood supply to the right lower extremities. Subsequently, a laparotomy was performed by colorectal surgeons. A fixed and solid tumor was observed at the right inguinal fossa, involving the external iliac artery, external iliac vein, ileum, and right obturator lymph nodes. En bloc tumor resection was successfully performed including a partial resection of the external iliac artery, external iliac vein, and ileum (Figure 4). The postoperative course was uneventful except for a prolonged ileus. She was discharged home 30 days after surgery. The leg edema was improved over time and did not recur during the follow-up.

The pathological evaluation revealed moderately and poorly differentiated adenocarcinoma consistent with rerecurrence of appendiceal cancer. Tumor invasion was extended to the external iliac vein with tumor embolus inside. The external iliac artery and ileum were free of tumor invasion but strongly attached to the tumor. The obturator lymph nodes and those in the resected mesentery of the ileum were positive for metastasis. Microscopically, cauterized cancer cells were present at the resection margin (R1 resection).

She received a FOLFIRI regimen as adjuvant chemotherapy. After six courses, a follow-up CT scan showed liver metastasis, para-aortic lymph node metastasis, and peritoneal dissemination. These recurrent lesions were considered unresectable. The chemotherapy regimen was altered to trifluridine/tipiracil. After seven courses, a follow-up CT scan showed a significant progression of the liver metastasis. Regorafenib was then administered for two weeks; however, it was discontinued because she denied further aggressive treatment. She had received palliative care and died one and half years after the last surgery.

## 3. Discussion

Curative surgical resection is crucial for the improved survival of LRCC. A systematic review concerning outcomes of resection for LRCC demonstrated that the R0 resection rate was 51%, and a five-year survival after R0 resection of 52% compared to 11% and 0% for R1 and R2 resection, respectively. They concluded that resection of LRCC is performed safely with long-term survival but that patients who could undergo resection had good performance status; therefore, the results may have been influenced by patient selection [9].

Several studies have investigated the prognostic factors after surgery for LRCC by univariate and multivariate analysis. These factors include margin status [6, 7, 10, 11], number of LRCC [6, 7, 10], site of LRCC [6, 10], presence of distant metastasis [6, 7], age, stage of primary tumor, preoperative carcinoembryonic antigen (CEA) level [6], pathological grade, largest tumor diameter [7], time to first recurrence [10], nodal involvement of primary tumor, and vascular invasion of LRCC [11]. The locoregional recurrence rate of colon cancer is 4.4% to 12.8% [2–5]. The risk factors for locoregional recurrence include the stage of primary cancer [2–5], location of primary cancer, emergent surgery [2, 3], lymph-vascular invasion [4, 5], bowel perforation [3, 5], bowel obstruction, margin involvement, and local tumor invasion [5].

LRCC often invades other organs such as the bladder, uterus, ovaries, and small intestine. Previous reports have shown that 26–100% of patients with LRCC undergo multivisceral resection [6–8, 11, 12]. In this case, the recurrent tumor involved the external iliac artery, external iliac vein, right obturator lymph nodes, and the small intestine. Before surgery, with careful assessment of the radiologic findings of the tumor extent, we consulted cardiovascular surgeons for reconstructive surgery involving the external iliac vessels. We suggest that preoperative consultations and discussions with surgical teams are extremely important for oncologic multivisceral resection for LRCC. Some authors suggested that resection of recurrent colorectal cancer with vascular involvement is contraindicated [13, 14]. Although there are limited numbers of publications, some authors suggested that aggressive resection of recurrent colon cancer with vascular excision is safe, contributing to longer survival and better quality of life [15–18]. For instance, Abdelsattar et al. reported 12 cases of surgery for LRCC involving the aortoiliac axis. They reported that seven of 12 patients who underwent arterial reconstruction had no graft complications, an R0 resection rate of 58%, and a four-year overall survival rate of 55% [19].

Currently, many patients receive systemic therapy including chemotherapy and radiotherapy before and/or after surgical resection for recurrent colorectal cancer. One advantage of preoperative chemotherapy is that we can evaluate the biological aggressiveness of metastatic colorectal cancer and choose appropriate patients who would receive long-term oncologic benefit from surgical resection [20]. In this case, the patient received multiple regimens of chemotherapy combined with the surgeries. She had pre- and postoperative mFOLFOX6 for the first tumor recurrence. Then, she had pre- and postoperative bevacizumab and/or FOLFIRI for the second tumor recurrence. For the recurrence after the last surgery, she sequentially received trifluridine/tipiracil and regorafenib. We used bevacizumab as molecular-targeted agents because of the KRAS mutant status. Some studies have advocated neoadjuvant therapy for colorectal cancer using bevacizumab [21–23]. These reports concluded that neoadjuvant chemotherapy with bevacizumab is oncologically feasible with good response, increased tumor resectability, and improved long-term survival.

Some authors have reported the effect of surgical resection combined with systemic therapy on LRCC. Harji et al. showed that preoperative chemoradiotherapy and/or chemotherapy for LRCC improved the radical resection rate [11]. Ohira et al. reported 19 cases of neoadjuvant chemotherapy, and Hallet et al. reported 15 cases of neoadjuvant chemoradiotherapy followed by radical resection for LRCC [12, 24]. Despite the small numbers of cases, both reports demonstrated that neoadjuvant treatment increased the curative resection rate with long-term survival.

However, the efficacy of systemic therapy on survival remains controversial. Kogler et al. reported that neoadjuvant or adjuvant therapy may influence median survival, although there was no statistical difference [8]. In contrast, Akiyoshi et al. and Bowne et al. showed that pre- and postoperative chemotherapy and radiotherapy for LRCC had no influence on disease-specific survival [6, 7].

In this case, we initially expected that the treatment strategy with sequential chemotherapy followed by surgical resection for LRCC would have provided long-term oncologic benefit. She survived two years and nine months since the diagnosis of rerecurrence. Because the rerecurrent tumor was locoregional, she might have survived for similar duration without surgical resection. Before the last surgery, she had suffered from leg edema, and symptomatic relief was achieved by the surgical resection. Afterwards, she never suffered from leg edema. Therefore, we assume that the surgical resection may have contributed to improved quality of life and better tolerability for the subsequent chemotherapy, rather than oncologic benefit.

The pathological evaluation revealed that cauterized cancer cells were present at the resection margin (R1 resection). Intraoperative radiotherapy with surgical resection has been shown to be beneficial for locoregionally advanced primary or recurrent colorectal cancer and other malignancies [25–27]. In our institution, intraoperative radiotherapy is not available; however, postoperative radiotherapy may have been another treatment option considering the resection margin was histologically positive.

Recent reports have shown the efficacy of en bloc resection of primary pelvic tumors involving main iliac vessels [28, 29]. These reports suggested that surgery was feasibly performed in specialized institutions. To our knowledge, this is the first report that exhibited surgical resection with vascular reconstruction for recurrent appendiceal cancer following systemic chemotherapy.

There are concerns about the choice of vascular graft and the need for venous reconstruction during the surgery. In this case, we used a synthetic graft for arterio-arterial interposition. Autologous grafts are preferred in exenteration surgery where operative field may be contaminated by concomitant bowel resection [30]. We performed a femoro-femoral arterial bypass before laparotomy; therefore, we thought that there was less risk of graft infection. Tsukushi et al. compared the results between the patients of arterial reconstruction alone and those of arteriovenous reconstruction in limb-salvage surgery for soft tissue sarcoma [31]. They concluded that the results did not indicate the usefulness of additional venous reconstruction after vascular resection in the lower extremity.

Systemic therapy for LRCC appears to contribute to the curative resection rate and long-term survival in selected patients. However, there are no generally accepted protocols for systemic therapy for LRCC. Further investigations including randomized trials are needed to elucidate a standardized treatment strategy for LRCC.

## 4. Conclusion

We reported a case of locoregionally recurrent appendiceal cancer in which our multimodal treatment provided relatively a long survival after the diagnosis of recurrence. Multiple regimens of neo- and adjuvant chemotherapy with surgical resection contributed to the oncologic outcome. Patient selection with careful assessment, availability of appropriate expertise, and meticulous discussions is necessary for the optimal treatment of LRCC.
